# The psychological effects of protective isolation on haematological stem cell transplant patients: an integrative, descriptive review

**DOI:** 10.1007/s00520-025-09186-2

**Published:** 2025-01-31

**Authors:** Rachel S. Lee, Lesley E. Halliday

**Affiliations:** Faculty of Medicine, University of Nottingham, Nottingham, UK

**Keywords:** Protective isolation, Stem cell transplant, Haematological malignancy, Patient care

## Abstract

**Purpose:**

Protective isolation is used during haematopoietic stem cell transplantation (HSCT) to protect patients at increased risk of infection. However, it is suggested that the intensity of strict isolation conditions combined with intense treatments can impact patients psychologically. This review explored the psychological effect of protective isolation on HSCT patients.

**Method:**

CINAHL, MEDLINE, and ASSIA databases were used to search for qualitative research undertaken between 2016 and 2023. Quality was appraised using the CASP tool and thematic analysis was utilised to identify themes using Thomas and Harden as a guiding framework.

**Results:**

Five papers were included and demonstrated that being in protective isolation during HSCT hospitalisation and after discharge created a feeling of disconnection from others and society, and that long periods of contemplation and a feeling of loss of control led to negative psychological impacts. All included papers found that patients experienced a range of negative emotional states during their time in protective isolation.

**Conclusion:**

Psychological health management is an important part of holistic patient care. Patients who experience HSCT report considerable negative psychological effects from the need for protective isolation. Interventions and strategies to improve this are slow to be developed and have not received the necessary focus in recent years. Critically, to maximise the patient experience and provide the best care possible, interventions are urgently required to minimise the longer-term psychological impact of HSCT in this patient group to contribute to maximising quality of life post-HSCT.

## Introduction

Haematopoietic stem cell transplantation (HSCT) is a complex and specialised medical procedure. It is used to treat haematological malignancies which are now the 5th most common cancer in the UK. Approximately 37,320 cases are diagnosed every year and 1 in 19 people will be diagnosed with a haematological malignancy at some point during their life [1]. Between 2006 and 2019, the average annual number of HSCTs increased by 5% per year [2]. HSCT involves suppressing the immune system through ‘conditioning therapy’ (chemotherapy and possibly total body irradiation) to minimise the risk of healthy new stem cells being rejected by the body’s immune system. However, this does increase the risk of infection and can lead to neutropenic sepsis, which is the prominent cause of death for patients receiving HSCT.

Consequently, protective isolation is used for infection control during HSCT. Protective isolation typically involves single-occupancy rooms for strict isolation and the restriction of visitors, and when discharged, standard practice requires patients to continue rigorous self-isolation for at least 3 months [3, 4]. The intensity of this isolation in addition to treatment effects makes HSCT immensely challenging for patients.

It has been suggested that 20% of patients who experience ongoing health challenges, such as a haematological disorder or malignancy, have an increased risk of developing depression [4]. This probability may be increased during HSCT due to the strict conditions of protective isolation and inadequate and controlled interaction with friends, family, and staff. There is also a concern that mental health issues may affect a patient’s health and recovery. This may potentially lead to longer stays in hospital and further costs to healthcare [5]. After HSCT, patients were found to be at greater risk of negative psychological effects due to low satisfaction with visiting hours and not receiving adequate emotional support [6]. When asked about visiting hours, most patients wanted less restrictions and the ability to spend more time with their loved ones. However, in contrast, other patients chose not to socialise with visitors [7] as they perceived family and friends as a cause of further distress and so preferred to be alone. Importantly for most patients, their emotional well-being depended on contact with friends and family [8]. Nevertheless, visitors are prospectively a source of infection as pathogens have been identified in the hands of visitors and emphasised the need for immunocompromised patients to have restrictions on visitors [9, 10].

## Methods

### Search strategy

This integrative, descriptive review followed the five stages proposed by Whittemore and Knafl [14]: namely (1) problem identification, (2) literature search, (3) data evaluation, (4) data analysis, and (5) presentation. An integrative review is a specific review method that allows for the inclusion of a range of methodologies and combines empirical or theoretical literature to provide a comprehensive understanding of a particular phenomenon of concern. This review aimed to be as comprehensive as possible. The review focused on qualitative studies—those that use open-ended techniques, such as interviews and non-statistical techniques for analysis as qualitative studies allow concepts to be evaluated in context (e.g. how the experiences of patients in protective isolation impact psychological health). Searches were completed between November 2022 and March 2023. After an initial scoping search, inclusion and exclusion criteria (see Table 1 in the Appendix) were developed to assist with refining the search. Using these criteria, a search of academic databases including CINAHL, Proquest, Medline, and ASSIA was performed using the keywords HSCT, protective isolation, psychological effects, and synonyms haematological stem cell transplant, bone marrow transplant, BMT, psychological impact, and emotional impact (both independently and combined using Boolean operators, such as ‘and’). Parameters of the search strategy were a date restriction from 2016 to 2023, research written in English, and included published peer-reviewed papers. Reference lists provided further sources of information through cited articles and books authored prior to 2016. These were included only if the title focused on psychological challenges that HSCT patients may face or added benefit or clarity to the review.

### Study selection

**Fig. 1:**
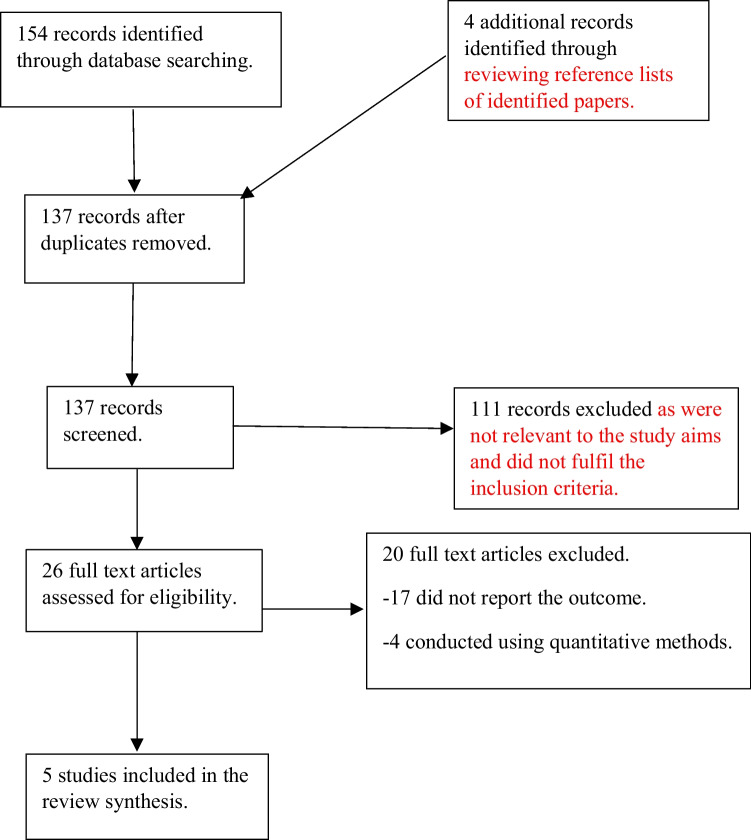
PRISMA flowchart

Screening of research gathered from the search was completed using the PRISMA (Preferred Reporting Items for Systematic Reviews and Meta-Analyses) [15], which also enabled the different phases of the search, application of inclusion/exclusion criteria, omission of unsuitable papers, and final included studies to be clearly documented [16] (see Fig. 1).

### Quality appraisal and data extraction

The final included papers were reviewed for quality using the CASP (Critical Appraisal Skills Programme) qualitative checklist [17]. The CASP tool consists of 10 questions: 9 addressing quality and 1 addressing ‘value’ (contribution to existing literature). All studies used a qualitative approach due to the focus of the research being on patient experiences and were considered to be of mixed quality by both authors (see Table 2 in the Appendix). Study risk of bias was also assessed using CASP and each author assessed each study independently and in duplicate with disagreements resolved through consensus.

## Findings

The initial search strategy identified 154 records. The PRISMA flowchart (see Fig. 1) demonstrates the process used to exclude studies. Five studies remained and were used in this review. The findings of the primary papers are summarised in tabular form with descriptions of the key characteristics of each (see Table 3 in the Appendix).

The five studies included all used qualitative methods [19–23]. The studies were carried out in Italy [19], Australia [20], Iran [21], the USA [22], and China [23]. There were no studies identified that have been completed in the UK. Four of the studies involved semi-structured interviews with 60 patients who had received HSCT treatment [18, 21–23]. One study was conducted by a survey completed by 441 patients [20]. Two of the interviews were carried out by nurse researchers [19, 23], one was by a trained psychiatrist [22], and one was by the author who had 10 years’ experience within the subject area [21]. One study focused on patients throughout treatment [22]. The other four studies were carried out post-HSCT [19–22]. Two studies explored the impact of protective isolation during HSCT [19, 23]. The other three studies explored the overall impact of HSCT treatment [20–22].

Inductive thematic analysis was performed following the three stages described by Thomas and Harden [18]. The first author applied the process outlined by Thomas and Harden and identified themes based on the psychological effects of protective isolation in HSCT. The second author independently reviewed each paper assessing for common themes, and after discussion, the final four themes were agreed upon: (1) feeling disconnected, (2) contemplation, (3) loss of control, and (4) negative emotional states.

### Theme 1: feeling disconnected

All five studies demonstrated how being confined to a room with limited contact resulted in a feeling of disconnection from others and society [19–23]. This resulted in patients experiencing immense loneliness during their time in the hospital and afterwards in the community: *The isolation is heavy, because you are actually alone and you lack support* [19]. Loneliness was often felt after just a few days of isolation [23] and the mental pain of being lonely and homesick was identified as being even less bearable than the physical pain: *I felt that the physical pain was bearable. However, the mental pain and stress broke me down. I’m so lonely and so homesick* [23].

Patients also expressed feeling disconnected from society. They reflected on how their social world had diminished due to the physical restrictions of isolation [20]. In addition, it was identified that protective isolation created a feeling of alienation from other human beings and suppressed emotions which would normally be shared with others: *After the transplantation I felt that I have become another human being and I should suppress these emotions* [21].

Protective isolation gave some patients a sense of safety claiming that being disconnected from the outside world also protected them from its demands, making them feel safe and even described being alone as a salvation: *Being in isolation is a salvation, because you could not cope being with others* [19]. However, in contrast, other patients felt unsafe and frightened. They perceived the isolation as a *dangerous trap* or as *being imprisoned* which contributed to the negative emotions they experienced [22].

### Theme 2: contemplation

Spending long periods alone in protective isolation provided many patients with time to spend in deep, reflective thought [19, 21–23].

During protective isolation, HSCT patients felt discouraged and uncertain about their treatment and whether it would be successful and fear about cancer recurrence: *Tremendous fear that the cancer would come back* [22] and the inability to return to their pre-cancer level of functioning [21]. Thinking about, and fear of death was also common: *I thought that I would die. I thought that it would end like that, as I was so bad!* [19]. Overall, it was acknowledged that fear and uncertainty are two main emotions experienced by patients during protective isolation [23].

It needs to be acknowledged though, that, contemplation during protective isolation, for some patients, produced positive psychological effects. Patients stated they felt lucky to be *given a second life* and were grateful for the care they received [19]. Other patients developed *strong positive emotions* due to recovery and sensing that *new life had begun* [23].

### Theme 3: loss of control

Four papers revealed that patients experienced a loss of control during protective isolation. This may be a loss of identity due to the implications of being in isolation, a loss of their old selves before treatment, or a feeling of *losing one’s mind* [19, 20, 22, 23].

During HSCT treatment and recovery, patients lost the capacity to work, became reliant on others, and lost their independence. This led to a loss of self-worth. Patients stated that they noticed large changes in their personalities. They also *felt like a non-entity* [20], felt they had lost their old selves and needed to rediscover who they were. This feeling of powerlessness and loss of control was heightened due to the added restrictions and isolation: *I did not expect it to be so difficult to feel so powerless* [22].

Patients also experienced cognitive symptoms during isolation, such as confusion and aprosexia. The confusion was facilitated by intense treatment and anxiety and this often prevented planned isolation activities to be completed. One patient expressed that they were unable to use the Wi-Fi stating, *it was impossible because I was completely out of my mind* [19]. Other patients reported symptoms of aprosexia and memory loss. The physical, emotional, and cognitive symptoms experienced by patients during isolation were unpredictable and changed throughout the treatment process suggesting a loss of control [22].

### Theme 4: negative emotional states

All studies revealed that patients experienced a range of negative emotional states including guilt, anxiety, and depression [19–23].

Patients stated how family and friends played a large part in providing emotional support throughout treatment. However, this could have a terrible impact on loved ones causing huge disruption to their lives. Patients often felt guilty [20] and a ‘burden’ [22] due to how their illness and treatment had impacted their family. These feelings were amplified for patients who had children*: I couldn’t see my daughter and be close to her. That hurt me most* [19].

Stress and anxiety were commonly experienced and severity increased with the time spent in isolation [23]. In addition to feeling anxious about treatment and future outcomes, patients felt stress and worry about loved ones [19]. Protective isolation was seen to increase the level of this stress. Anxiety was also experienced about the transition from being in isolation in the hospital, to being at home and having reduced restrictions: *Well, I’ve just been anxious to getting back to being allowed to do things* [22].

Depression was experienced and increased with time spent in isolation: *Now I feel like time is getting longer and longer, and I feel very depressed and lonely* [23]. Patients felt that depression had an impact on their interaction with their family or carers. Some patients chose to have no interaction with their loved ones or the outside world: *I couldn’t even Facetime with them [family] because I was so depressed* [22]. Depression had a varying impact on recovery, function, and quality of life throughout HSCT [22].

## Discussion

All five of the studies identified patients feeling disconnected from others and society [19–23]. This feeling of disconnection resulted in extreme loneliness after only a few days in isolation [23] and mirrors findings in other studies [14, 24].

To maintain social effectiveness [20, 21], stay connected to society, and receive necessary social support, it is important for patients to maintain relationships with others [14]. These may be loved ones [25], members of staff, peer support [26], or volunteers [27]. However, it has been acknowledged that some patients are more at risk of having problems in maintaining relationships during isolation. These include being a male patient, patients with lower education levels, those with higher levels of pain post-transplant, those having had fewer chemotherapy cycles before HSCT, and those with low satisfaction with visiting hours [28]. This suggests that it is necessary to ensure that all HSCT patients have access to ongoing social support and access to enhanced psychological services during protective isolation to mitigate and minimise these recognised [29].

Safety was also identified as an issue for patients in isolation [19, 22] both negatively experienced as imprisonment and positively experienced as an enhanced feeling of protection due to the sterile conditions and the private environment. Earlier literature found that being cared for in a single bedroom enabled patients to feel secure and able to focus on themselves and their recovery [20]. It may also help to make improvements to the isolation room itself to make it more comfortable and homely for the patient. Art interventions, such as the study *Open Window*, have been shown to have a positive influence on the patient experience and QoL through enhancing the environment and providing stimulation [31].

Protective isolation provided patients with time to contemplate on their present circumstances and future lives [19, 21–23]. Patients were found to either spend time feeling fearful and uncertain or were enabled to find ways of feeling positive and hopeful. Previous research reported patient’s experiences of fear during HSCT and the significant influence of the fear of recurrence on patient’s psychological well-being and quality of life [32]. This was magnified in female patients and those with pre-existing subclinical depressive symptoms [33]. Various activities have been suggested to help occupy and refocus patient’s thoughts to more positive areas including therapeutic music videos [34] and creative arts [35, 36].

Nonetheless, contemplation is important and required to enable some patients to regulate their own thoughts and feelings and to enable effective adaptation to the isolation environment [14]. It is suggested that for effective coping, patients are required to find meaning in the experience—this meaning making is considered essential in HSCT to minimise the psychological impact of HSCT isolation and assist patients in finding positive personal growth [37].

Experiencing loss of control of both identity and minds was often reported [19, 20, 22, 23]. After HSCT, patients return home to live very different lives to pre-treatment. They are unable to socialise and return to work for some time. It is evident that this leads to a loss of independence, identity, and self-worth [28]. Other studies have discussed the challenges post-HSCT has regarding career and financial domains [38] and how this affects the ability to provide family support leading to a sense of uselessness [39].

A review which examined the effects of isolation on memory found that social isolation combined with loneliness had an adverse effect on cognitive functioning [40]. It suggested that cognitive symptoms reduce confidence in the ability to manage other symptoms experienced during HSCT, thus affecting physical and psychological well-being of patients [41].

Negative emotional states, such as guilt, anxiety, and depression, are commonly reported as part of the HSCT protective isolation patient’s experience [19–23] and referred to as *isolation as a source of suffering* [14], linking psychological distress to both the emotional and physical demands of isolation. Other studies provide evidence that protective isolation can lead to depression [42, 43] and anxiety [44, 45]. In fact, it has been posited that 20% of patients develop a psychological disorder [46] with female patients being more at risk [43]. Pre-transplant psychosocial issues also predict patients experiencing depression and poor quality of life during isolation [42], highlighting a need to be aware of the patient’s mental health before transplant to predict those at increased risk of psychological issues especially given that pre-transplant depression and anxiety may decrease a patients’ overall survival time after transplantation [47].

Evidence from the wider literature, combined with the findings from this review, acknowledge that there are considerable psychological effects of being in protective isolation for patients during HSCT. Findings have enabled several suggestions to improve the experience of patients in protective isolation post-HSCT. These include pre-HSCT mental health assessments to identify patients at risk, increasing staff knowledge of the potential psychological impacts, clear referral pathways if required, and increased support and education for family and loved ones of HSCT patients.

## Conclusion

This review explored research on the psychological impact of protective isolation for HSCT patients. It addressed the need for an increased focus on this area of care after HSCT, particularly in the UK where research is lacking, as outlined by the Clinical Commissioning Policy on HSCT [2]. It compared findings to an earlier review [12] and made recommendations for improving the management of patients’ post-HSCT experiencing protective isolation to contribute to an improved quality of life.

This review had several limitations. There was a paucity of available research that focused on the psychological impact of protective isolation in HSCT. This is not surprising due to the limited patient population and their vulnerability which may have affected participation in research. This resulted in only five studies being included in the thematic analysis. Sample sizes were also small in the reviewed studies, although this is not unusual for qualitative methods and only three databases were searched meaning quality studies could have been missed. Importantly, no appropriate studies were found based on patient experiences in the UK. However, recommendations may still be useful and provide insight into enhanced practice in the NHS and UK healthcare delivery around improving the patient experience. Nevertheless, the findings from all studies were complimentary and, when taken together, have provided useful insight into the psychological experience of HSCT patients. It demonstrated that there are considerable psychological effects of being in protective isolation during HSCT and made recommendations for future management to minimise the impact on patients.

Due to the increasing number of HSCTs being carried out in the UK, it is essential that more research is conducted that focuses on this impact of treatment and patient management. Further research on protective isolation in the hospital and at post-discharge for patients in the UK is urgently required as currently there is little evidence available to inform effective patient support or best practice care within the context of the NHS. Additionally, more research focus is required on the psychological impact of protective isolation. This should cover all aspects of mental health conditions, including the impact on cognitive function. A previous review, which was conducted 8 years ago, identified similar psychological effects impacting HSCT patients, suggesting that little progress has been made to improve the management of these symptoms or improve the patient experience. HSCT is undoubtedly a necessary treatment approach for haematological disorders but when combined with the treatment process, symptoms produced, and protective isolation it is an extremely difficult and intense experience.

## Data Availability

The authors declare that all data supporting the findings of this review are found within the article and its supplimentary material.

## Appendix

See Tables 1, 2 and 3.

**Table 1:** Inclusion and exclusion criteria

	**Inclusion**	**Exclusion**	**Reason**
Who?	Adult HSCT patients in protective isolation	Children and non-HSCT patients	The patient group is relevant to adult nursing degree
Where?	Countries using protective isolation for HSCT patients	Countries not using protective isolation for HSCT patients	Focal point of research topic
Language	Papers published in English	Papers not published in English	To save time as easily read and understood
When?	Papers published since 2016	Papers published before 2016	Based on a similar review by Biagioli et al. [11]. The extended literature review will detect any variations or improvements in care in the last 7 years
Text	Full text only	Abstract	Entire article can be reviewed
Research type	Qualitative Primary research	Quantitative Secondary research, e.g. systematic reviews	Topic is concerned with patient’s experiences and emotions
Type of studies used	Interviews Focus groups Open-ended questionnaires	Random Control Studies	The author of the study has engaged with the participants and collected data
Information sources	Primary research	Secondary research, e.g. systematic reviews	Based on the original material
Type of data	Empirical research	Opinion pieces and discussion papers	The author has observed and gained experience of the patient’s feelings and views

**Table 2:** Quality assessment using the CASP tool for qualitative research

Included study	Is there a clear statement of the aims?	Is the method appropriate?	Is the recruitment strategy appropriate?	Has the relationship between researcher and participants been considered?	Were ethical issues considered?	Was the data analysis sufficiently rigorous?	Are the findings clear?
Biagioli et al. (2016)	*Yes*—to explore the lived experience of isolation	*Yes*—phenomenological design, semi-structured interviews	*Yes*—purposive sampling	*Yes*—by 2 nurse researchers not involved in care	*Yes*	*Yes*	*Yes*
Brice et al. (2016)	*Yes*—to gain an understanding of the impact HSCT has on quality of life	*Yes*—phenomenological design, survey with only 1 broad question	*Yes*—purposive sampling, recruitment over the phone	*Not stated*	*Not stated*	*Not stated*	*Yes*
Nasrabadi et al. (2019)	*Yes*—to explore experiences of patients post-HSCT	*Yes*—phenomenological design, semi-structured interviews, three broad questions	*Yes*—purposive sampling, but only 6 participants	*Yes*—conducted by the author with experience of the subject	*Yes*	*Yes*	*No*
Amonoo et al. (2020)	*Yes*—to explore psychological distress post-HSCT	*Yes*—phenomenological design, semi-structured interviews	*Yes*—purposive sampling, but asked during HSCT hospitalisation	*Yes*—by trained psychiatrist with no previous relationship with participants	*Not stated*	*Yes*	*Yes*
Chen et al. (2022)	*Yes*—to explore symptom experience and self-management strategies of HSCT patients during hospitalisation	*Yes*—phenomenological and longitudinal design, semi-structured interviews	*Yes*—purposive sampling	*Yes*—by nurse researcher	*Yes*	*Yes*	*Yes*

**Table 3:** Included studies

Included study	Country	Sample	Phenomenon	Data collection method	Outcome
Biagioli et al. (2016)	Italy	10 allogeneic HSCT patients	The lived experience of isolation	Interviews	The general theme found was identified as being isolated to achieve transformation. Sub-themes included (1) the special place for transformation, (2) the experience of embodied transformation, and (3) light and shade from inside and outside
Brice et al. (2016)	Australia	441 allogeneic HSCT patients	The impact HSCT has on patient’s quality of life	Questionnaire	The HSCT survivor’s world and their opportunities had diminished compared with their life prior to the onset of the disease. Themes included the following: the failing body and diminished physical effectiveness, the changed mind, the loss of social connectedness, the loss of the functional self, and the patient for life
Nasrabadi et al. (2019)	Iran	6 HSCT patients	The lived experiences of Iranian patients post HSCT	Interview	Participants experienced isolation from social, occupational, and emotional activities, and excessive control by family members
Amonoo et al. (2020)	USA	25 allogeneic HSCT patients	The psychological distress after transplantation	Interviews	Negative emotional experiences reported were feeling trapped, fear, guilt, discouragement, and powerlessness. Patients reported that these interfered with motivation to participate in health behaviours important to the transplant recovery
Chen et al. (2022)	China	19 HSCT patients	Symptom experience and self-management strategies of HSCT patients during hospitalisation	Interviews	HSCT patients experienced a complex and dynamic array of symptoms from admission to discharge They experienced the dual forces of internal predicament and external strength in symptom self-management during hospitalisation
